# Implementation of a magnet hospital model: attracting and retaining healthcare staff in a Swedish hospital

**DOI:** 10.1108/JHOM-04-2024-0159

**Published:** 2024-09-03

**Authors:** Peter Nilsson, Maria Gustavsson

**Affiliations:** Department of Behavioural Sciences and Learning, Linkoping University, Linkoping, Sweden

**Keywords:** Magnet hospital model, Organisational change, Implementation, Employee resourcing

## Abstract

**Purpose:**

Staff shortages in the healthcare sector increase the competition for qualified staff. A magnet hospital is intended to attract, and retain healthcare professionals. This article aims to investigate the challenges related to implementation of a magnet hospital model, and given these challenges, to analyse the interplay between different organisational levels in a Swedish hospital.

**Design/methodology/approach:**

The data collection followed the implementation of a magnet hospital model and consisted of 14 meeting observations, 31 interviews and 13 document analyses.

**Findings:**

The model implementation was driven by a top-down approach, with accompanying bottom-up activities, involving healthcare professionals, to ensure adaption to the hospital’s conditions at different organisational levels. The findings revealed that the model was more appealing to top management, seeking a standardised solution to attract and retain nurses. Clinic managers preferred tailor-made solutions for managing their employee resourcing challenges. Difficulties in translating and contextualising the model to the hospital’s conditions created challenges at every organisational level. Some were contained within a level while others spread to the organisational level below and turned into something else.

**Originality/value:**

Apart from unique empirical material depicting the implementation of a magnet hospital model as an effort to attract and retain healthcare professionals, the value of this study lies in the attention given to the challenges that arise when responsibility for implementing a management model is shifted from top management to change agents tasked with facilitating and executing the organisational change.

## Introduction

Staff shortages are a major challenge in the healthcare sector. Employers are forced to compete for attracting and retaining qualified healthcare personnel. In parallel, there has been an increase in the tendency for healthcare professionals to change employers more frequently in search of more promising career opportunities, higher salaries and better working conditions (Clark *et al.*, 2006). Beechler and Woodward (2009) argue that this competition has sparked a global war for talent, fuelled by a global shortage of nurses and physicians. Before the Covid-19 pandemic, the World Health Organisation estimated that millions of people would need to be recruited by 2030 to fill the global staff shortage in the healthcare sector (WHO, 2016).

Scholars therefore argue that global competition makes it imperative for employers to develop strategies and models to attract and retain healthcare professionals (Steijn and Knies, 2021). One such proposed solution in the healthcare sector is the magnet hospital model (Svensson *et al.*, 2024; Wolf *et al.*, 2008). A magnet hospital refers to an organisation that, like a magnet, attracts and retains nurses despite the demand exceeding the supply of competence in the labour market (McClure, 2005; McClure and Hinshaw, 2002). Magnet hospitals are also associated with providing a high-quality patient care (Aiken, 2002; Kramer and Schmalenberg, 2008). Hospitals adopting the magnet hospital model can be categorised into “Capital M” and “Little M” types (McClure, 2005). Capital M hospitals have received recognition through the American Nurses Credentialing Center (ANCC) and achieved certification, while Little M hospitals have not sought certification but implemented a selection of the ANCC criteria for a magnet hospital.

This article reports on the implementation of a magnet hospital model in a Swedish university hospital. Its aim was to address the problem of recruiting and retaining nurses and other healthcare professionals. The hospital’s top management decided to tailor a magnet hospital model (Little M) to their local conditions and involve all levels of the organisation. For two years, we followed the implementation of this magnet hospital model from its initiation at the top management level to its end at the clinic level, and the challenges that different actors (management, operations developers, human resource practitioners and healthcare staff) faced along the way. Thus, this article aims to investigate the challenges related to implementation of a magnet hospital model, and given these challenges, to analyse the interplay between different organisational levels in a Swedish hospital.

The following section outlines previous research on the magnet hospital model and theories of organisational change. This is followed by a description of the research settings and the method. The findings are then presented in the next section. The final section provides a discussion and conclusions.

## Previous research on the magnet hospital model

The term magnet hospital originated during the 1980s, when McClure *et al.* (1983) investigated, on behalf of the American Nurses Association (ANA), what led some hospitals in the United States to recruit and retain more registered nurses than others despite a nationwide nursing shortage. This study became the starting point for further research on magnet hospitals (Havens and Aiken, 1999; McClure and Hinshaw, 2002). Studies have defined the “magnetism” of these hospitals as comprised of three “ingredients” **(**McClure *et al.*, 1983**),** 14 “forces” **(**Urden and Monarch, 2002**)**, eight “essentials” **(**Kramar and Schmalenberg, 2005a**)** or five “components” (Wolf *et al.*, 2008) (Table 1). According to Kramer and Schmalenberg (2005b), the structure-process-outcome perspective captures what a magnet hospital is**:** the existence of certain structures (working conditions) enables certain processes (attract and retain), resulting in desirable outcomes in terms of quality of patient care.

Accordingly, magnet hospitals are associated with the attraction and retention of nurses and the quality of patient care (Aiken, 2002; Kramer and Schmalenberg, 2008). In terms of attracting and retaining nurses, magnet hospitals are associated with high job satisfaction (Kramer and Schmalenberg, 2005b; Rodríguez-García *et al.*, 2020), lower burnout levels (Kelly *et al.*, 2011; Rodríguez-García *et al.*, 2020), a safer work environment (Clarke *et al.*, 2002; Rodríguez-García *et al.*, 2020) and low staff turnover (Rodríguez-García *et al.*, 2020; Staggs and Dunton, 2012). Nurses in magnet hospitals report less intention to leave the organisation (dit Dariel and Regnaux, 2015; Kelly *et al.*, 2011) and**,** conversely, more intend to remain in the profession than nurses in non-magnet hospitals (Lacey *et al.*, 2007). Despite this, McClure (2005) argues that staff stability is not an explicit criterion for the hospitals that apply to the ANCC to be certified as magnet hospitals. Regarding patient outcomes, magnet hospitals are associated with higher patient satisfaction (Scott *et al.*, 1999) and lower patient mortality (dit Dariel and Regnaux, 2015; Rodríguez-García *et al.*, 2020). Nurses in magnet hospitals also rate the quality of patient care as high (Kramer and Schmalenberg, 2008). Good patient outcomes can, in turn, improve attractiveness and thereby help to retain nurses.

The magnet hospital model spread early to the United Kingdom (Aiken *et al*., 2008; Buchan, 1994) and has since spread to other countries (Anderson *et al.*, 2018; Svensson *et al.*, 2024). The international experiences indicate that there are challenges in the translation and contextualisation of the model. The magnetic elements identified in Table 1, for example, can be incompatible and not balanced for positive organisational effect (McClure *et al.*, 1983). There is also a conceptual ambiguity regarding what characterises magnetic work environments (Kramer and Schmalenberg, 2005b). This has called into question the validity and reliability of magnet hospital studies (McClure, 2005).

For example, McClure *et al.* (1983) original study did not use a representative sample of the total number of hospitals in the United States. This, and other research issues, suggest that drawing conclusions about why some hospitals are better than others in attracting and retaining nurses is more difficult than first believed (Havens, 2001). According to Scott *et al.* (1999), it cannot be ruled out that non-magnet hospitals have the potential to exhibit the same positive characteristics as magnet hospitals, or that there may be factors, other than those identified in the magnet schemes, that are more important in explaining successful nurse recruitment and retention. Trinkoff *et al.* (2010) show that the working conditions of nurses in magnet and non-magnet hospitals differ little, while other studies indicate that non-magnet hospitals can have higher staffing levels and produce better patient outcomes than magnet hospitals (Goode *et al.*, 2011). Literature reviews highlight the difficulty of establishing causality (dit Dariel and Regnaux, 2015; Rodríguez-García *et al.*, 2020). The impact of magnet certification on patient and nurse outcomes remains uncertain because research on magnet hospital models is contradictory. Despite the uncertainty, the magnet hospital model continues to spread and be implemented by organisations in different countries.

## Organisational change theory

In this article, implementation is assumed to be a form of planned change, that is, a deliberate attempt to redefine an organisation’s current state to move it toward a desired future (Stouten *et al.*, 2018). To achieve a planned change, top-down or bottom-up approaches, or a combination of these two, called integrated, can be used. However, these approaches differ in terms of change initiation and change execution (Heyden *et al.*, 2017). The top-down approach is a planned and structured way to change that can be organised in different ways. Traditionally, this approach is initiated by top management, mediated through some form of collective change agency group or unit (Wylie and Sturdy, 2018) and executed by managers and employees in their workplaces, although sometimes reluctantly. In this approach, top management uses their formal position of power to realise their ambitions and plans. This “trickling-down” of change responsibility assumes that it is possible to predict and control the change process and its outcome, and that the process will be a linear, sequential journey towards a desired future state. The bottom-up approach, on the other hand, is initiated and executed by middle managers and employees in their respective workplaces. This type of change is employee-driven, occurring mostly incrementally and resulting in improvements of operations, processes and routines at the workplace level. This approach assumes employee participation and learning opportunities to achieve successful change (Nilsen *et al.*, 2020).

With an integrated approach, the strengths of the top-down and bottom-up approaches are combined. For example, employee participation and learning can be integrated into the sequential, linear change process to ensure that the organisation reaches its desired goal. Previous research has shown, however, that all change approaches have flaws: only about a third of organisational change initiatives actually succeed (Stouten *et al.*, 2018). This low success rate cannot entirely be attributed to the choice of change approach. It is much more a result of how change agents in an organisation, both individually and collaboratively, interpret and manage the change process (Heyden *et al.*, 2017; Wylie and Sturdy, 2018) and the nature of the challenges that can arise during a change process (Kotter, 2008; Stouten *et al.*, 2018). Change agents at different organisational levels can therefore interpret and manage the same change initiative differently. For a change process to be successful, change agents need to agree on the goal they are all working towards and the means for solving the challenges that may arise.

## Research settings

This study was carried out at an urban university hospital in Sweden. In this northern European welfare state, the regional authorities provide tax-financed public health care for all inhabitants within specific catchment areas. The hospital employed approximately 7,000 employees and provided assessment, treatment and care in all medical specialties, 24 h a day, all year round. The management hierarchy consists of a top management level, production unit managers at the middle level and clinic and ward managers at the workplace level, all of whom have responsibility for employee resourcing. Like many other hospitals in Sweden, this hospital struggled to attract and retain employees, particularly it had a high turnover rate among registered nurses. In response to the staffing challenge, the hospital’s top management decided to adopt an employee resource strategy based on the magnet hospital model and four magnet areas were selected to be implemented: leadership, professional development, quality of care and innovation. The HR director held overall responsibility for this large-scale implementation, while the operational responsibility was delegated to a project management team (PMT) consisting of a project manager and two HR strategists. The PMT’s task was to facilitate and effectuate the implementation and report back to the HR director after one and a half years within the two-year project period. Initially, the PMT invited healthcare professionals to participate in four workgroups aimed at clarifying and contextualising the selected magnet areas. They then proceeded to anchor the implementation of this model at the production unit and clinic levels.

The studied clinic chose to implement the hospital’s magnet model. The clinic provided highly specialised planned and emergency surgical care. It had 180 employees of which nurses made up the largest professional group, followed by assistant nurses and physicians. For several years, the clinic had struggled with high staff turnover and difficulties in recruiting and retaining nurses in particular. At the time of the study, there were approximately 25 nursing vacancies.

## Method

### Design and selection

This study adopted a case study design **(**Yin, 2018**)**, to which it applied an interactive research approach (Ellström *et al.*, 2020). The management of the hospital contacted the researchers to discuss the idea of following the implementation of a magnet hospital model adapted to the hospital’s conditions. Once an agreement had been reached between the researchers and the hospital’s PMT a plan was drawn up to enable the implementation to be studied. Since the implementation process was followed iteratively over a two-year period, the selection of study participants was carried out successively, depending on the direction the implementation took.

### Data collection

Following an interactive research approach, the data collection chronologically followed the implementation of the magnet hospital model, from its beginnings with top management and its progress, via the production unit, to the clinic level. Therefore, the data collection method was not decided at the beginning of the study but adapted to each stage of implementation. As such, a mix of data collection methods were used. In total, the data consists of 14 meeting observations, 31 semi-structured interviews and the analysis of 13 documents (Table 2).

An open-descriptive technique inspired by meeting ethnography (Sandler and Thedvall, 2017) was used for the documentation of the 14 meetings observed. At the top management level, five workgroup meetings were observed. These meetings were attended by 59 healthcare professionals, mainly nurses, who discussed the magnet areas: leadership, professional development, quality of care and innovation. The participants met two or three times over a two month period in four workgroups, one for each magnet area. Each meeting lasted about two and a half hours. The researcher who attended the meeting used a laptop to document conversations, questions and proposed solutions for the magnetic area that was under investigation in each workgroup. Meetings of all four workgroups were observed, and one group’s meetings twice, but we were not able to participate in all meetings as some were held in parallel. At the production unit level, nine three-hour meetings where observed. At these meetings the PMT met with representatives from the production unit and clinic levels, to anchor the implementation of the magnet hospital model.

A total of 31 interviews were conducted with representatives from the three organisational levels to gain deeper insight into the implementation process (Table 2). All interviews were conducted individually at the representatives’ workplaces and by both researchers, with each session lasting approximately 60 min. Semi-structured interview guides adapted to the representatives at the three levels were used. At the top management level, the four PMT interviews covered three themes: prerequisites for implementing a magnet hospital model; the implementation process and its results. At the production unit level, the interviews with four operations developers (OpDev) and six HR practitioners (HR) covered four themes: knowledge and familiarity with the magnet hospital model; implementation of the magnet hospital model at the clinic level in daily work; support needed to implement the magnet hospital model and the magnet hospital model’s potential improvements and organisational effects.

At the clinic level, three interview guides were developed to carry out a total of 17 interviews with two clinic managers, 13 nurses and two assistant nurses. At this level, the primary problem of attracting and retaining staff was focused because few interviewees were aware that the hospital was implementing a magnet hospital model. All three interview guides although slightly adjusted to match the different professions, covered three themes: clinic and workplace features and conditions; workplace changes made to attract and retain healthcare staff; and intentions to leave the clinic. The interview guides provided a structure for the discussion of topics, but the conversational format encouraged the interviewees to talk openly about their experiences. All interviews were digitally recorded and then transcribed in their entirety.

In addition, 13 strategically selected documents such as reports, minutes of meetings and guidelines were collected to supplement our meeting observations and interviews (Table 2). These documents have mainly been used to describe the research setting and the implementation of the magnet hospital model.

### Data analysis

All the data material has served as a basis for analysing the implementation of the magnet hospital model. A thematic analysis, inspired by Braun and Clarke (2006), was conducted, aiming to identify, discern and analyse recurring themes. A first reading of the entire data set – field notes of meetings, interview transcripts and documents – provided an overall picture of the implementation challenges. Then, an inductively driven coding process was conducted to identify repeated patterns of meaning (themes) and to cluster, review and name the challenges that arose at different organisational levels during the implementation of the magnet hospital model (see Table 2).

### Ethical consideration

The research meets the ethical guidelines of the study country including adherence to EU:s General Data Protection Regulation (GDPR). All participants were informed verbally and in writing of the aim of the study and all gave their informed consent before participating. The participants understood that they could withdraw their participation at any stage of the study without giving any reason. To guarantee confidentiality we have not disclosed specific details about the participants or the hospital studied.

## Findings

### Challenges at the management level

The implementation of the magnet hospital model (hereinafter referred to as the model) was initiated by the top management, but the PMT was given the operational responsibility to carry out the implementation. The first challenge that arose was the PMT’s ambiguous mandate to drive the implementation process at the production unit and clinic levels as the responsibility for employee resourcing was delegated and dependent on decision-making at these levels. Without formal authority for the PMT to put pressure on the other organisational levels to adopt the model, questions were soon raised about the PMT’s mandate and ability to effect change. The PMT found it difficult to bear responsibility for the implementation because it had to be adapted to all organisational levels. The unclear mandate was openly discussed by the PMT.

Sometimes I’ve felt that someone at top management level is needed, who can come and sort of take responsibility for the decision. (PMT)

One OpDev from the production unit explained that the reason behind the PMT’s unclear mandate could be its fuzzy mission statement, which lent itself to varying interpretations of their task.

There must be clear governance and a clear mandate: what is the purpose, where are we going, why are we doing this? (OpDev5)

When the PMT faced questions about the purpose of the implementation it became evident that the fuzzy mission statement could be open to different interpretations. Some felt the current staffing situation was a matter of employee resources versus quality of care, while others wondered whether a single model could add anything new to the efforts to accommodate healthcare professionals with diverse employment preferences.

And if this activity does not lead to concrete measures that make us act in a different way than we already do today, then this is a total waste (OpDev5).

The fuzziness made it difficult to unite the professions in joint implementation work. To remedy this, the PMT undertook several activities to anchor the model and to engage staff in the change process. Healthcare professionals were invited to participate in four workgroups aimed at clarifying the four magnet areas, which were compiled into a set of guidelines for the continued implementation. However, this initiative, along with other anchoring activities was met mostly with negative reactions throughout the implementation process. The PMT found managing the persistent resistance to change much more challenging than expected. The resistance was multifaceted, with nurse representatives and members of the nurses’ national interest organisation questioning the inclusion of professions other than nurses in the model. Involvement by physician in the change process was minimal, which was seen as passive resistance to the nurse-linked model.

I don’t think we can go to the physicians and say that now we are going to work with the magnet hospital model, you must deal with it in a different way. (HR5)

Also, the resistance to change pushed the PMT to address structural resistance linked to the choice of the top-down implementation strategy. This challenged the existing distribution of staff responsibilities, with some decisions being made by top management while others were decentralised to production units and clinics. The PMT was also obliged to deal with a more general resistance to change projects, as many staff viewed the implementation of the model as yet another attempt by top management to introduce undesirable management models.

People are tired of projects and new things that are unexpectedly introduced and become a little averse to these things. (HR9)

The PMT believed that, given these challenges, completing the implementation in 18 months would be a challenge, especially given the scale of the process and unpredictable staff resistance.

I thought from the beginning that it would not be like that, that the process would take so long, that I instead would be able to work at the production unit level as a starter in some way, or a facilitator or something. But … it didn’t turn out that way. (PMT)

Despite the challenges, the PMT continued to arrange workshops and meetings to inform and support the handover of the implementation of the model to the production unit and clinic levels. However, despite its efforts to sustain the implementation, the PMT was advised by representatives for the production unit and clinics to revisit the implementation plan. But the plan was not revised since the HR director terminated the PMT’s assignment after 18 months. During the following 6 months at the end of the project, clinic and ward managers were responsible for implementing the model. Also, they were allowed to adopt their own interpretation of its meaning and the extent to which they intended to use it.

### Challenges at the production unit level

At this level, the OpDevs and HRs had a key role as the facilitators supporting clinic managers in the implementation of the model. The introduction of the model had led to discussions about who was formally in charge of staffing. This posed challenges for the OpDevs and HRs who had to figure out their roles and responsibilities in the change process. The dilemma was that the production unit was self-governing with delegated staffing responsibilities. When the model was proposed, staffing issues were moved to the PMT for centralized decision-making. This created confusion and led to speculation about whether implementing the model was voluntary.

I interpret it as not voluntary, that everyone has to work on it, but how much you work on it is up to you, which actually means that you can do something very small to make a big project out of it. (HR3)

These ambiguities led to 18 months of discussion until the HR director finally clarified that the production unit had the independent authority to decide on staffing, including the adoption of the model. However, the difficulties OpDevs and HRs had translating the model and providing support to clinical-level managers slowed down the implementation. Both OpDevs and HRs voiced their uncertainty about the way forward and the most effective implementation strategy to support the clinic managers. Therefore, they advocated for a revised strategy that actively involved clinic managers and other professions in the implementation process.

Ward managers and clinic managers need to be on this train, I mean, this isn’t something that I or we in the production unit management can introduce ourselves if we don’t have participation from the clinics and wards. (HR4)

The OpDevs and HRs requested more explicit support from the PMT to help them involve managers and healthcare staff more actively in the implementation work. Also, they adviced the PMT to avoid using the term “magnet hospital model” at the clinic level. The term “model” had become a negative buzzword in the hospital, widely associated with negative experiences of previous top-down change attempts.

I think it’s the label that makes it difficult … (OpDev7)

Another concern facing OpDevs and HRs was the model’s primary association with the nursing profession. They feared that the other professions would exclude themselves or not feel involved in the implementation process. An additional challenge arose from varying and sometimes conflicting understandings of the primary focus of the model: was it predominantly centred on employee resourcing or the enhancement of quality of care? Since both OpDevs and HRs were tasked with supporting clinic managers, their diverging interpretations of the implementation’s main purpose made it difficult for them to align their efforts towards a coherent direction of change. With implementation depending on the interconnections between OpDevs and HRs, it was important that they reached some sort of agreement.

We’re like a team that works together, and we need to discuss, who does what, should we work together with certain parts and so on. (HR2)

Agreeing on their collaboration was easy, but agreeing on a single standardised model to address the hospital’s extensive challenge of attracting and retaining healthcare staff proved more challenging.

I think that there are different magnets if you are a physician or if you are a healthcare administrator. And now we’ve built a model that should be generic for everyone, which means that it might be unsatisfactory for everyone too. (OpDev7).

The OpDevs and HRs perceived a problem–solution mismatch; the solution provided by the model could not sufficiently address all employee resourcing needs because it did not consider local conditions within clinics or the needs of the different professions. The challenge was also that some clinics had started to use variants of the original magnet hospital model, and they wanted to maintain these variants to attract and retain staffs. This meant that some clinics chose to develop their own strategies to remedy staff shortages according to their local conditions.

### Challenges at the clinic level

The clinic followed in this project chose to implement the model. During their inplementation process, there were several challenges identified that were similar to those at the production unit level. One such challenge concerned the limited leeway the clinic manager had to influence the clinic’s employee resourcing. She had no authority to decide on important factors known to attract and retain nurses, such as salary levels or working time models. The model did not include these issues, as they were reserved for the top management to decide.

I only know that the PMT has said no to everything and … I think that’s bad. I think there will be times when you must think differently. And I think there are many healthcare professionals who work in other clinics that we could attract back if we started listening to what people say they need. (Clinic manager)

The clinic manager appreciated that the adoption of the model had been made optional, but the model could not guide her in the struggle to balance the demands of reactive and proactive staffing strategies. Critical staff shortage could often occur at short notice. During such circumstances, the clinic manager had to reduce the number of beds, move patients to other wards, use agency nurses or order the clinic’s nurses to work overtime. The nurses we interviewed expressed great dissatisfaction with the clinic’s reactive staffing policy, which had going on for years.

When things are brought to a head, then the managers wake up and quickly talk about “It can’t be done without you” and “You’re so good” and “You can’t disappear from here”. But by then it’s gone too far. Managers should have seen these signals much earlier and acted and encouraged individuals before they ended up in the situation “Now I’m quitting”. (Nurse2)

Another challenge was that the model did not give any guidance on how to handle the frequent turnover of nurses. The clinic manager pointed out that it had a “blind spot” as it did not focus on preventing the outflow of skilled nurses. The clinic had struggled with this situation in the past but had largely been unsuccessful in retaining nurses. According to the nurses, colleagues resigned due to long-term dissatisfaction with working conditions. Some of the nurses expressed their intention to leave the clinic, while others had already submitted their resignations.

I’m still thinking about resigning. I’m not going to work at the clinic for the rest of my life, and that end point is in the relatively near future. (Nurse3)

I’ve submitted my resignation due to the working conditions. I feel like I can’t stay. (Nurse9)

Dealing with the high turnover of nurses was a top priority for the clinic. It prompted the clinic manager to focus on the magnet “professional development” as a way to attract and, above all, retain nurses.

Since I took over as the clinic manager a year and a half ago, this has been the clinic’s highest priority, because we’ve had a lot of problems with losing people, especially nurses. (Clinic manager).

The nurses stressed that having opportunities for professional development was a prerequisite for staying at the clinic. Both the nurses and assistant nurses felt that in the first year of their employment, there had been plenty of learning opportunities. However, in the years thereafter, further professional development often required them to organise learning opportunities themselves outside the clinic.

However, I feel that I’ve seen a lot and learned a lot even though I’ve been loyal to the same clinic. I have a colleague today who’s in surgery because there’s something she wants to see there that she hasn’t seen yet … she wants to see it hands-on, so she’s observing a surgery today. … there’s no one saying, “No, this isn’t possible”. Instead we manage it ourselves and we give ourselves a couple of hours so we can go and be part of it. (Nurse5)

The quote indicates that opportunities for professional development gradually changed from being regular to something that nurses and assistant nurses organised themselves as part of their everyday work.

## Discussion

This article provides insights into the challenges related to the implementation of a tailored magnet hospital model to a hospital’s conditions. The implementation of the model had mainly **a** top-down approach (Wylie and Sturdy, 2018), focused on deliberately developing the attractiveness of the hospital to attract and retain healthcare professionals. With inspiration of the magnet hospital model, the top management implemented its change initiative via the mediation of the HR director, the leadership of the PMT and the specific actions carried out by local change agents (Heyden *et al.*, 2017) such as the OpDevs and HRs**,** and clinic managers at the production unit and clinic levels. There was consensus at all levels that the hospital needed to change the way it attracted and retained healthcare professionals. At first glance, the change process appears to be sequential and linear, running from top to bottom. However, the implementation also had bottom-up elements (Nilsen *et al.*, 2020), especially once the PMT recognised the necessity to anchor the model in the hospital’s operations and involve staff.

During the implementation, several challenges arose that caused the process to take different turns. Some of these challenges were linked to a particular organisational level, while other challenges filtered down from one level to the next and came to affect the implementation work of others. This meant that a challenge either could “stay there”, or be “pushed down” to the next level, when fuzzy mandates and directives left room for interpretation and created difficulties for change agents in translating the model.

At the management level, the PMT encountered challenges such as an unclear mandate, a fuzzy mission, a time-limited assignment and resistance to change. The findings show that the lack of a clear mandate and the short timescale made it difficult for the PMT to realise the implementation of the model and involve healthcare staff. There are two other reasons why the PMT had difficulties in implementing the model. First, they had to work with top management’s decision to organise the implementation as a project, as a parallel structure running alongside the formal organisation. This implied that, although the PMT had the responsibility for implementation, they never had the authority to force the other levels to adopt the model. The PMT, therefore, acted as a change unit that was not only temporary but also separate from the self-governing production unit and the lines of management stretching down to the clinic level. This kind of organisation of change is not uncommon but, as Wylie and Sturdy (2018) argue, it can be met with reluctance to change in the workplace. This is what happened; the PMT met resistance to the implementation of the model.

The second reason why the PMT had difficulties implementing the model was because resistance to change was activated early in the implementation process**,** and the PMT never had the chance to manage it constructively. A profession-based resistance was demonstrated actively by nurses and passively by physicians. Resistance was also demonstrated externally by the nurses’ national interest organisation. A cultural resistance was manifested amongst the staff at large, as many used the implementation to revive their negative experience of previous attempts at top-down change. Structural resistance arose from the self-governing autonomy that the production unit had over employee resourcing. The findings indicate that as soon as the HR director announced the end of the project, the balance tipped in favour of forces opposing the adoption of the hospital’s magnet model.

The challenges that began at the management level as “troubles for the PMT” then filtered down to the production unit level. The production unit had to deal with problems pushed down onto them, but these problems could manifest in other forms that gave room for interpretation but also uncertainty. The production unit was in charge of staffing, but there was considerable uncertainty about the way forward, with conflicting interpretations of directives and a problem-solution mismatch. The findings indicate that this challenge was a consequence of ambiguity at the upper level. Because of a lack of clarity at the top level, OpDevs and HRs could not fully understand their role or execute their responsibilities during the implementation. However, they were collectively identified as the key change agents; they were trusted to bring about the implementation of the model at the production unit level and further expected to interpret it to provide support for clinic-level managers. As such**,** they were expected to come to an agreement on the goal (Kotter, 2008; Stouten *et al*., 2018) regardless of the conflicting interpretations which had arisen as implementation progressed. The cause of these conflicting interpretations can be found in the original magnet hospital model’s duality of focusing on: the attraction and retention of nurses, and the quality of patient care (Aiken, 2002; Kramer and Schmalenberg, 2008). The findings show that this requirement posed a challenge for OpDevs and HRs. It made it necessary for them to engage healthcare professionals other than nurses in implementing a nurse-associated model while aligning their joint efforts towards one specific aspect of the model: employee resourcing. Adapting one model to meet the employee resourcing needs for all clinics within the production unit seemed to be a difficult task. As the findings indicate, this challenge was filtered down to the clinic level where it proved to create several challenges.

At the clinic level, the challenge of implementation was mainly about employee resourcing. Clinic managers had limited leeway to influence their clinic’s staffing levels. They were caught between their existing reactive staffing approaches and the model’s purported usefulness in addressing the negative turnover of nurses. At this level, adoption of the model was voluntary; the clinic studied knew it needed to improve its attractiveness. When conditions known to attract nurses, such as higher salaries and reasonable working hours, could not be controlled, this clinic chose to adopt only one magnetic element: professional development (see Table 1). It was argued that better training opportunities would reduce the high level of nurse turnover by mitigating dissatisfaction with working conditions (dit Dariel and Regnaux, 2015; Rodríguez-García *et al.*, 2020). In the magnet hospital model, attracting and retaining skilled nurses are essential phases in the strategy to secure a stable employee base. However, in other models of employee resourcing, an additional phase that must be considered to comprehensively grasp the sequential process of managing employee resourcing is the out process (Wallo *et al.*, 2016). This marks a blind spot within the magnet hospital model. It does not provide a mechanism for handling mass voluntary nursing departures, especially when leaving has reached such a point that it is almost too late to take measures to retain nurses. A lack of awareness of local conditions in the clinic meant that the complete magnet model could not be implemented.

### Study limitations

One limitation is that the quality of care in the studied magnet hospital model (see for example Aiken, 2002; Kramer and Schmalenberg, 2008), is not investigated. However, the workgroup discussions aiming to clarify the four magnet areas did discuss the quality of care. In the data collected, interest in quality of care as a magnetic element faded as the need to attract and retain healthcare staff became a more pressing issue during the implementation. It is, of course, possible that workplaces that attract and retain nurses also provide a high quality of patient care. Another limitation is our decision to only follow one clinic. Other clinics might have adopted the full model or other magnetic elements, especially when the use of the model became voluntary. Despite that, the strength of the study lies in its relatively extensive data material, which enabled a reconstruction of the implementation of the hospital-adapted magnet hospital model across all organisational levels.

## Conclusion

The hospital’s intention was to implement a hospital-adapted magnet hospital model to strengthen its ability to attract and retain healthcare professionals. This large-scale implementation was designed as a top-down approach but with bottom-up elements involving different healthcare professionals and three hierarchical levels, to ensure that it would be adapted to the hospital’s local conditions. Based on the findings, the first conclusion is that a top-down organisational change approach like this, even if it did include bottom-up elements, takes a long time and has a high risk of failure. The magnet hospital model appealed more to top management, who sought a standardised solution, while clinic managers preferred tailor-made solutions adapted to their local conditions. This can be interpreted as a conflict between two contrasting solutions for addressing the problem of employee resourcing, which is rarely optimal.

The second conclusion is that challenges arose at each level of implementation due to the difficulties in translating and contextualising the model for the hospital’s different level-specific conditions. The filtering of challenges downwards from one level to the next, revealed a pattern where challenges which persisted within a certain organisational level were then spread to the level below, and at those levels sometimes turned into something else. Understanding the implementation challenges that can potentially emerge for top management and change agents tasked with leading, facilitating and executing the plan is essential for organisations to achieve their desired objectives.

To uncover the implications of the implementation of a magnet hospital model more thoroughly, two aspects require further research: the effectiveness of the magnet hospital model as an employee resourcing strategy; and the effectiveness of an organisational change strategy that involves all organisational levels, from top and middle to clinic, especially given the challenges that can interplay between them during an implementation process. The study bridges the gap between organisational change theory and practice, highlighting the utility of understanding implementation challenges at different organisational levels. Ultimately, this knowledge can have an impact on hospitals’ employee resourcing and quality of patient care.

## Figures and Tables

**Table 1:** A comparison between the magnet categories of different sources

McClure *et al.* (1983)	Urden and Monarch (2002)	Kramar and Schmalenberg (2005a)	Wolf *et al.* (2008)
Three ingredients of magnetism: (1) Administration: Management style, Quality of leadership, Organisational structure, Staffing, Personnel policies (2) Professional practice: Quality of patient care, Teaching, Image of nursing (3) Professional development: Orientation, Inservice and continuing education, Formal education, Career development	14 forces of magnetism: (1) Quality of nursing leadership (2) Organizational structure (3) Management style (4) Personnel policies and programs (5) Professional models of care (6) Quality of care (7) Quality improvement (8) Consultation and resources (9) Autonomy (10) Community and the hospital (11) Nurses as teachers (12) Image of nursing (13) Interdisciplinary relationships (14) Professional development	Eight essentials of magnetism: (1) Working with other nurses who are clinically competent (2) Good nurse-physician relationship and communication (3) Nurse autonomy and accountability (4) Supportive nurse manager or supervisor (5) Control over nursing practice and practice environment (6) Support for education (7) Adequate nurse staffing (8) Concern for patient is paramount	Five magnetic components: (1) Transformational leadership (2) Structural empowerment (3) Exemplary professional practice (4) New knowledge, innovations and improvement (5) Empirical quality outcomes

**Source(s):** Authors’ own work

**Table 2:** Summary of (1) data-collection methods and (2) challenges at each of the three organisational levels during the implementation of the magnet hospital model

Top management level	Production unit level	Clinic level
(1) *Data collection methods*		
- 5 observations of meetings - 4 interviews with members of the project management team - 6 document analyses	- 6 interviews with HR practitioners - 4 interviews with operations developers - 9 observations of meetings - 7 document analyses	17 interviews in one clinic: - 2 managers - 13 nurses - 2 assistant nurses
(2) *Implementation challenges*		
- Unclear mandate - Fuzzy mission - Resistance to change - Time-limited assignment	- Who is in charge of staffing? - What way forward? - Conflicting interpretations of the model - Problem solution mismatch	- Limited leeway - Balancing reactive and proactive employee resourcing - The blind spot in the model

**Source(s):** Authors’ own work
